# Multi-Center Catalytic Oxidation of the Sotalol Drug Adsorbed on Gold Nanoparticles

**DOI:** 10.3390/molecules31101714

**Published:** 2026-05-18

**Authors:** Ekaterina A. Kolobova, Ksenia N. Makarova, Elena V. Solovyeva

**Affiliations:** Institute of Chemistry, Saint-Petersburg State University, Universitetskaya 7/9, 199034 Saint Petersburg, Russia

**Keywords:** gold nanoparticle, capillary electrophoresis, beta-blockers, surface reaction, nanozyme

## Abstract

Currently, gold nanoparticles are increasingly used in targeted drug delivery nanostructures. However, their intrinsic catalytic activity is often overlooked when using them as a carrier. In this study, the interaction between the sotalol drug from the beta-blocker family and gold nanoparticles was investigated using capillary electrophoresis and high-performance liquid chromatography. Both methods showed that sotalol undergoes catalytic oxidation on the surface of citrate-stabilized gold nanoparticles into three products. Together with a cleavage of the isopropyl group from the nitrogen atom, the oxidation at the hydroxyl group occurs with the formation of a ketone. Analysis of electropherograms showed 100% conversion of sotalol after 48 h of incubation at a surface coverage of 1.2 × 10^19^ molecules per m^2^. To examine the role of reactive oxygen species, the experiments were performed in oxygen-saturated and oxygen-deficient gold nanoparticle dispersions. The effects of radical scavenger additives and pH of nanoparticle dispersion were also assessed. The influence of surface ligands on sotalol conversion was studied using gold nanoparticles coated with thiols, surfactants, and polyelectrolytes. Based on comprehensive data, the mechanism of gold-nanoparticle-assisted multicenter oxidation of sotalol is proposed.

## 1. Introduction

One of the main objectives of modern pharmaceutics and nanomedicine is a creation of targeted drug delivery systems capable of minimizing the systemic toxicity and increasing the bioavailability of drugs. Among the variety of nanomaterials, gold nanoparticles (GNPs) attract a special attention from researchers due to their exceptional biocompatibility, chemical stability, and simple surface functionalization [1,2]. GNPs are considered a promising platform for the delivery of a wide range of therapeutic agents [3,4,5,6], from well-known cytostatics such as doxorubicin [7,8,9,10,11,12] and paclitaxel [13] to insulin [14,15,16] and nucleic acids [17,18].

Loading of therapeutic agents onto gold nanoparticles can be achieved in two main ways. A number of drug molecules can be directly attached to the GNP surface through physical adsorption, ionic interactions, or covalent bonding [19,20,21]. The simplest method consists of the addition of the drug to the nanoparticle dispersion and holding for a certain period of time for adsorption, followed by centrifugation to remove excess molecules which did not attach to the surface. For composite drug loads or those requiring better stability, the GNP surface must be functionalized. This typically involves the application of specialized coatings, such as polyethylene glycol [22,23,24,25], to improve biocompatibility, the conjugation of peptides [26] or antibodies [27] for cell targeting.

However, when utilizing gold nanoparticles, their intrinsic catalytic activity, particularly in the oxidation of biologically active compounds, must be taken into account. While previous studies have documented the GNP-mediated oxidation of various compounds, such as dihydronicotinamide adenine dinucleotide [28], sodium ascorbate, and hydroethidine [29], they have primarily focused on the generation of reactive oxygen species (ROS). Among a few studies addressing the GNPs’ oxidative effects on drug molecules, most focus on the photocatalytic degradation of antibiotics in the context of environmental pollutant elimination [30,31]. However, identifying unintended chemical transformations of adsorbed GNP drug molecules and elucidating a specific oxidative surface reaction mechanism are also of high significance, since pharmaceuticals often possess multiple redox-sensitive functional groups. Understanding surface reaction pathways is critical for developing robust targeted drug-delivery strategies.

In a recent study, it was demonstrated that the propranolol drug from the β-blocker family undergoes catalytic oxidation on citrate-stabilized GNP, yielding a metabolite with a cleaved isopropyl group [32]. This process occurs via a single-electron transfer mechanism and raises an important question: is the GNP-catalyzed N-dealkylation specific to propranolol, or does it extend to other molecules containing the same structural fragment?

To answer this question, sotalol, another β-adrenergic receptor blocker with an isopropyl group bonded to a nitrogen atom, was selected for the current study. We hypothesized that sotalol would undergo an analogous surface transformation, providing a deeper insight into the GNP-assisted N-dealkylation. In particular, confirmation of the general character of beta-blocker N-dealkylation catalyzed by GNP will entail a loss of their ability to stimulate beta-adrenergic receptors and therapeutic efficacy [33] when incorporated into hybrid nanostructures. The previously developed methodology of immersion experiments was applied, involving incubation of the drug in a GNP dispersion, centrifugation, and subsequent analysis of supernatant by capillary electrophoresis (CE) and high-performance liquid chromatography (HPLC).

## 2. Results and Discussion

**Figure 1:**
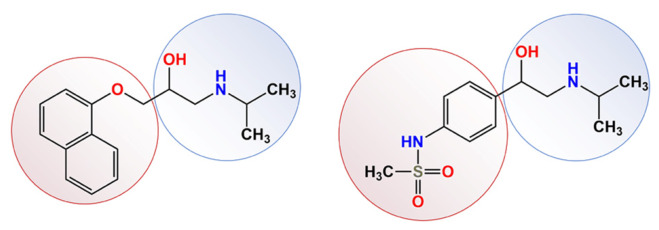
Comparison of the structures of two β-blocker drugs: propranolol (**left**) and sotalol (**right**).

A previous study of propranolol adsorption onto citrate-stabilized gold nanoparticles reported its oxidation into N-desisopropylpropranolol. To verify a general character of the discovered reaction, sotalol was chosen as another model β-blocker (Figure 1). Both compounds contain a propanolamine moiety (a secondary amino group with an isopropyl substituent and a hydroxyl group at the β-position), which is necessary for binding to β-adrenergic receptors. The key differences between the two molecules are the structure of the aromatic fragment and its bond with the propanolamine moiety: propranolol has a naphthoxy group, while sotalol has a phenyl with a methanesulfonamide group at the para-position.

### 2.1. Pro-Oxidant Effect of Gold Nanoparticles on Sotalol

**Figure 2:**
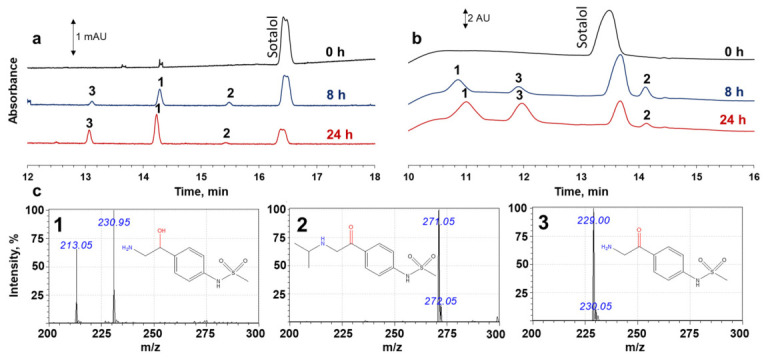
Electropherograms (**a**) and chromatograms (**b**) of supernatants after sorption of sotalol onto cGNP during 8 h and 24 h at pH 5.5; HPLS-MS spectra of oxidation products and their structures (**c**).

To verify the possible transformation of sotalol, the supernatants obtained after incubation in a citrate-stabilized gold-nanoparticle (cGNP) dispersion during 8 h and 24 h were analyzed by capillary electrophoresis. Three new peaks can be observed in the obtained electropherograms (Figure 2a): before sotalol, which appears as a broad peak of R- and S-enantiomers at 16.6 min, a strong peak arises at 14.4 min, a medium peak emerges at 13.2 min, and a small peak appears at 15.5 min. To identify the compounds responsible for new peaks, the same supernatants were analyzed by HPLC-MS (Figure 2b). The signals in the electropherograms and chromatograms were matched based on changes in peak relative intensities between the samples corresponding to the 8 h and 24 h sotalol incubation periods. Firstly, the most intense signals at an incubation time of 8 h were correlated: the peak in the electropherogram with a migration time of 14.4 min corresponds to the peak in the chromatogram with a retention time of 10.85 min. Since an area of the peak at 13.2 min increased with increasing sotalol adsorption time, while the peak at 15.5 min decreased in the electropherogram, the first signal corresponds to the peak at 11.90 min, and the second signal corresponds to the peak at 14.12 min in the chromatograms. All three products of sotalol surface reaction were identified using full scan mass spectra at chromatography scale: sotalol (t_R_ = 13.67 min) with *m*/*z* 273.05, product **1** (t_R_ = 10.85 min) with *m*/*z* 230.95, product **2** (t_R_ = 14.12 min) with *m*/*z* 271.05, and product **3** (t_R_ = 11.90 min) with *m*/*z* 229.05 (Figure 2c). The recorded mass spectra of the products **1**, **2**, and **3** differ from sotalol by 42, 2, and 44 *m*/*z*, respectively. Product **1** with *m*/*z* 230.95 is expected to correspond to N-desisopropylsotatol. Peak numbered **2** is a product of oxidation of the hydroxyl group. Product **3** is a compound with both a hydroxyl group and an amino group oxidized. Thus, in addition to N-dealkylation, sotalol undergoes oxidation at the hydroxyl group on the cGNP surface, yielding a ketone. This reaction is analogous to the previously observed oxidation of 1-phenylethanol to acetophenone, assisted by gold nanoparticles; see [34,35,36,37].

**Figure 3:**
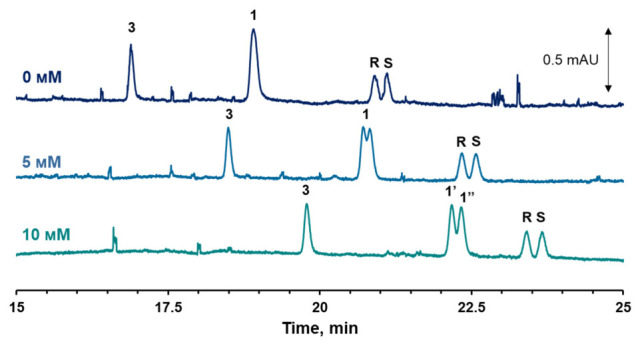
Electropherograms of supernatant after adsorption of sotalol onto cGNP for 24 h (pH 5.5) with different 18-crown-6 concentrations in the background electrolyte. Numbers 1′ and 1″ design R and S enantiomers of product **1**. Conditions: 100 mM FBS (pH 2); 25 mM HP-CD. Injection—2 s·50 mb. U = 20 kV; λ = 230 nm.

Products **2** and **3** are achiral compounds, while a stereogenic center is preserved in product **1**. Therefore, separating the product **1** enantiomers by capillary electrophoresis was important to corroborate the HPLS-MS results. Firstly, the concentration of HP-β-CD in the background electrolyte was increased up to 25 mM, but the enantiomer separation was not achieved. Secondly, 18-crown-6 was added to the background electrolyte (C = 5 mM) because it is capable of forming complexes with the protonated primary amino group of small molecules. Indeed, the separation of the enantiomers of product **1** was achieved, and the resolution improved with an increased concentration of 18-crown-6 in the background electrolyte (Figure 3). It should be noted that the enantiomeric ratio of sotalol, as well as product **1**, does not change, indicating the oxidation of the amino group is not stereoselective.

### 2.2. Influence of Adsorption Time on Sotalol Surface Transformations

**Figure 4:**
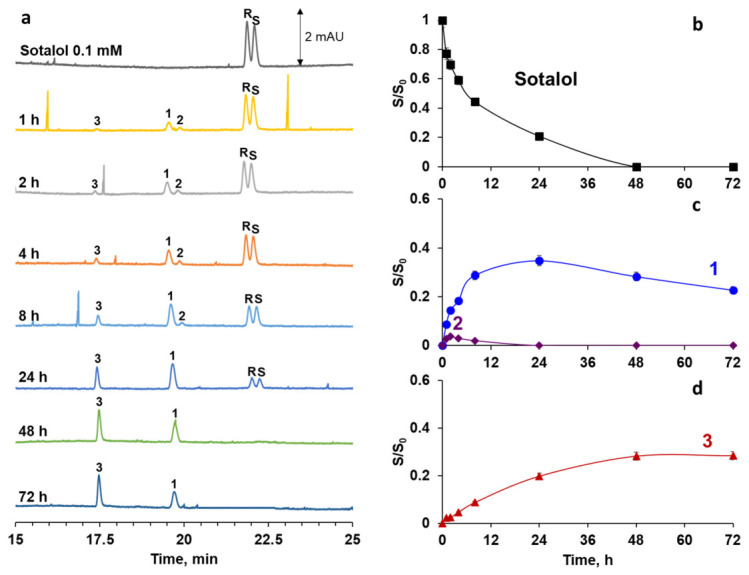
Electropherograms of supernatant after sorption of sotalol onto cGNP at different times at pH 5.5 (**a**); dependence of normalized peak area of sotalol (**b**), product **1** and product **2** (**c**), product **3** (**d**) on time of incubation.

Since sotalol undergoes cGNP-assisted transformation into several products simultaneously, the investigation of their accumulation as a function of adsorption time was a primary objective. The incubation period of sotalol in cGNP dispersion was prolonged up to 72 h. The time-dependent profiles were obtained using a normalized peak area *(S/S_0_)*, where S is the analyte peak area in the supernatant at a specific time point, and S_0_ is the initial peak area of a 0.1 mM sotalol solution (Figure 4). Product **3**, which forms as a result of N-dealkylation coupled with hydroxyl group oxidation, exhibited continuous accumulation over time. In contrast, the product of hydroxyl group oxidation (product **2**) reached its highest content at 4 h, while the N-dealkylation product (product **1**) reached its maximum at 24 h. After this time, the concentration of both products **1** and **2** gradually decreased. The signal from sotalol completely disappeared after 48 h, indicating its 100% conversion at a given ratio of the drug molecules to nanoparticle surface area (1.2 × 10^19^ molecules per m^2^). Based on the observed time-course trends, it can be concluded that products **1** and **2** gradually transform into product **3**, leading to an increase in its concentration after 48 h.

### 2.3. Influence of Environmental Parameters and Surface Chemistry on the Sotalol Transformations

**Figure 5:**
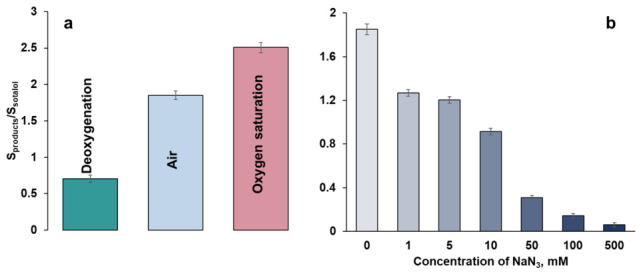
Histograms of the ratio of product sum areas to sotalol at different oxygen content (**a**) and concentrations of NaN_3_ (**b**) in immersion experiments at pH 5.5.

Since it was previously shown that oxygen participates in gold-nanoparticle-mediated N-dealkylation of propranolol [32], the influence of saturation of the cGNP dispersion with molecular oxygen on the oxidation of sotalol was also studied. For this purpose, the immersion experiments were carried out under deoxygenation and oxygen bubbling (Figure 5). Saturation of the cGNP dispersion with oxygen leads to an increase in the product–sotalol peak area ratio. Adsorption of sotalol under deoxygenation is followed by a decrease in the total area of the product peaks but does not exclude their formation (Figure 5a). This indicates that the oxygen chemisorbed on the gold surface, which is not removed by bubbling the cGNP dispersion with argon, participates in the reaction that is reasonably consistent with previous observations on transition-metal surfaces [38]. Additionally, sodium azide, a well-known radical scavenger, was added at different concentrations to the mixture of sotalol and cGNP (Figure 5b). In this case, the amount of the product decreases as the concentration of sodium azide increases. This is obviously due to a significant reduction in the concentration of ROS neutralized by azide. In addition, non-specific adsorption of azide-related species, which occupy a part of the available surface, cannot be excluded. Overall, it can be concluded that the oxygen adsorbed on the cGNP surface plays a key role in the sotalol oxidation.

**Figure 6:**
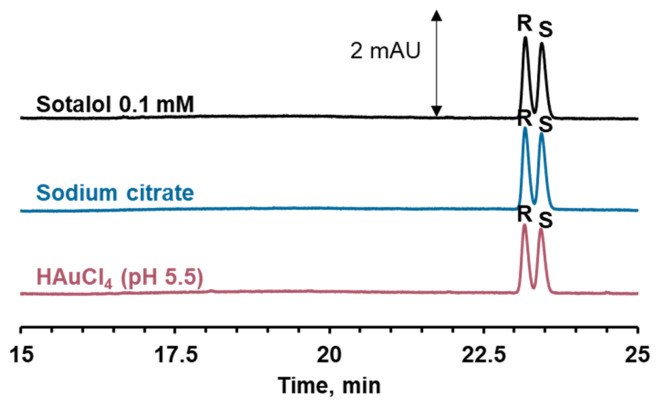
Electropherograms of supernatant after sotalol incubation in a solution of hydrogen tetrachloroaurate and a solution of sodium citrate for 24 h.

To verify the catalytic activity of gold nanoparticles, control experiments were performed using hydrogen tetrachloroaurate and sodium citrate solutions at the same concentrations as those used in the cGNP synthesis. These experiments showed no detectable sotalol oxidation products (Figure 6). This confirms that the observed transformation is catalyzed exactly by the gold nanoparticles, rather than by gold or citrate ions present in the cGNP dispersion.

**Figure 7:**
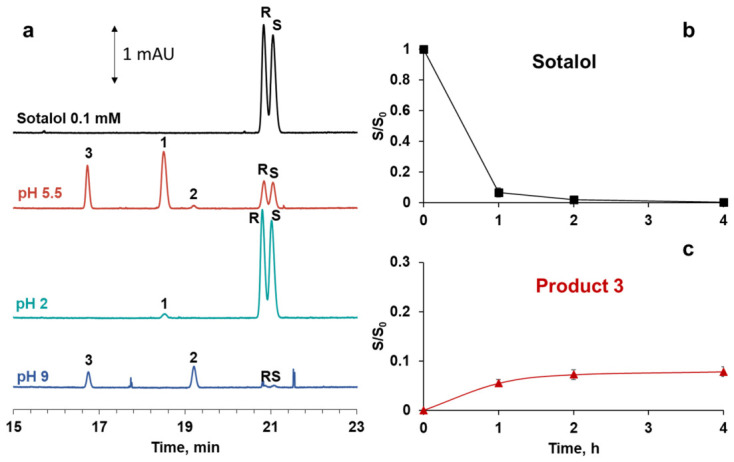
Electropherograms of supernatant after incubation of sotalol in cGNP dispersion at different pH levels (**a**); dependence of sotalol (**b**) and product **3** (**c**) content on incubation time at pH 9.

The pH level of the aqueous medium may significantly influence the sotalol oxidation on the cGNP surface. To verify this, immersion experiments were performed on highly acidic and basic cGNP dispersions. The total amount of adsorbed and converted sotalol was calculated from the peak areas in the corresponding electropherograms (Figure 7). At pH 2, the sotalol oxidation is almost inhibited, and the decrease in sotalol concentration in the solution is 0.8%. On the other hand, sotalol is rapidly oxidized in product **2**, which then transforms to product **3** at pH 9, resulting in a 97.6% concentration decrease. Under pH native for cGNP dispersion (~5.5), product **1** is a predominant product of sotalol oxidation, and the total amount of adsorbed and converted sotalol is 75.8%. The change in the yield of surface reaction products may be due to variations in the zeta potential of the nanoparticles and in the different charge states of sotalol. When the environment in the dispersion changes from slightly acidic (pH 5.5) to alkaline (pH 9), the magnitude of cGNP zeta potential decreases from −37 mV to −17 mV, which is accompanied by their partial coagulation. At a shift in pH from 5.5 to 2, the zeta potential changes insignificantly up to −32 mV. Thus, changes in GNP zeta potential cannot explain such a dramatic effect of pH on sotalol surface transformations. Obviously, the effect of pH is interrelated with the different ionization states of the sotalol molecule, which in turn affect its adsorption on the gold surface and the reaction mechanism.

**Figure 8:**
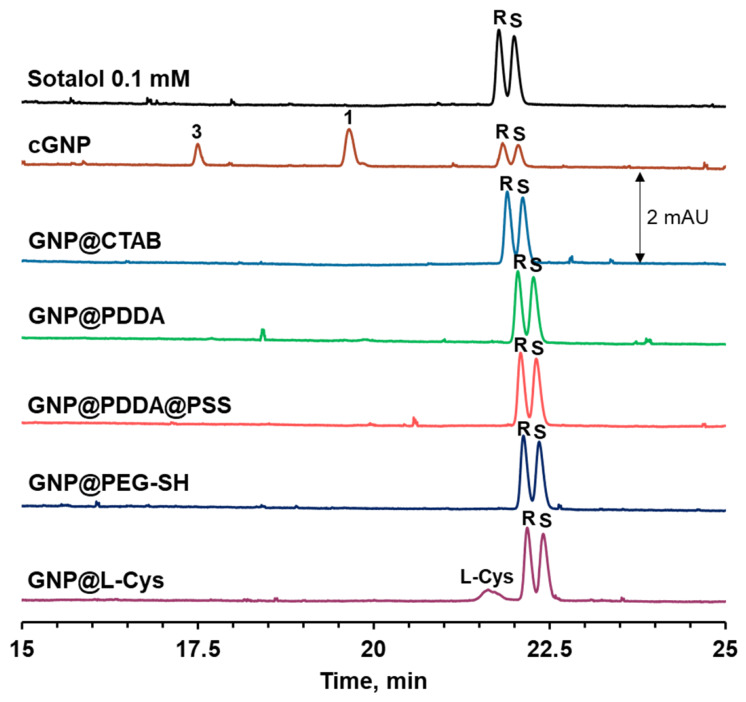
Electropherograms of supernatant after incubation of sotalol in GNP dispersions with different coatings.

The influence of GNP surface chemistry on sotalol oxidation was further investigated. Gold nanoparticles modified with different agents (L-cysteine, surfactant, and polyelectrolytes) were used. Regardless of the nature of GNP coating, a suppression of sotalol oxidation is observed (Figure 8). This can be due to several reasons. Firstly, the adsorption of sotalol is limited on the GNP with CTAB and PDDA shells due to electrostatic repulsion, as evidenced by the high positive values of their zeta potential (+27.4 and +43.2 mV, respectively) and by the observed constancy of sotalol concentration in the solution. Secondly, access of sotalol to the surface may be blocked on GNPs with L-cysteine and PEG-SH coatings due to the higher affinity of thiols to the gold surface. On the contrary, citrate ions on the surface of bare cGNPs are highly labile and can be easily replaced by many adsorbates, including sotalol. Thirdly, a suppression of ROS generation by GNP can also be caused by the coatings, which has already been observed before [39], along with inhibition of GNP catalytic activity in reduction reactions [40].

### 2.4. Mechanism of Sotalol Surface Transformations

**Figure 9:**
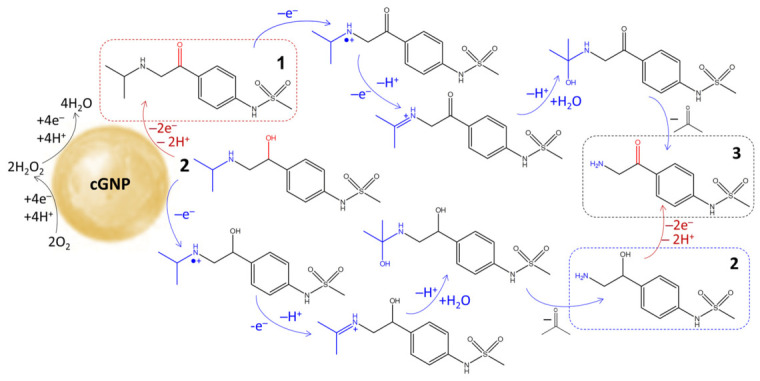
Proposed pathway of sotalol oxidation onto the surface of a gold nanoparticle.

Based on the experimental results, a dual-pathway mechanism of sotalol oxidation onto citrate-stabilized gold nanoparticles is proposed (Figure 9). Presumably, the process proceeds through a single-electron transfer mechanism, with the initial stage involving GNP forming a superoxide radical. This catalytic reduction of molecular oxygen to the superoxide radical is well established in the literature [41], including detailed mechanistic studies [29,42]. The interaction of sotalol with the superoxide radical proceeds via two parallel processes that affect different functional groups, both of which undergo further oxidation to form a single product. The oxidation of the hydroxyl group distinguishes sotalol from propranolol, in which it was not observed. The lack of reactivity in propranolol can be attributed to the structural environment of the propanolamine moiety. While sotalol has a conventional structure of substituted benzyl alcohol that plays a crucial role in stabilizing a transition state on the gold surface, the hydroxyl group in propranolol is a part of a more sterically crowded or electronically deactivated chain due to its attachment to the naphthoxy group.

The described transformation is supported by the results obtained at different pH levels. At highly acidic pH, the oxidation of the hydroxyl group is completely suppressed, while N-dealkylation occurs only to a minimum degree. The amino group is almost entirely protonated (pH < pKb), eliminating the neutral nucleophilic form required for the initial stage of isopropyl group cleavage. An increase in pH significantly accelerates the sotalol degradation and shifts the selectivity toward product **2**. The alkaline environment facilitates the deprotonation of the hydroxyl group into an alcoholate anion. As a superior electron donor compared to the neutral hydroxyl group, the alcoholate anion undergoes rapid oxidation.

## 3. Materials and Methods

### 3.1. Materials

To synthesize and modify citrate-stabilized gold nanoparticles, the following reagents were used: hydrogen tetrachloroaurate trihydrate (Aurat, Moscow, Russia), sodium citrate 5.5 aqueous (Vekton, St. Petersburg, Russia), L-cysteine (J&K Scientific, Beijing, China), cetyltrimethylammonium bromide (CTAB) (BLD Pharm, Shanghai, China), mercapto polyethylene glycol (PEG-SH), Mw ≈ 5000 g/mol (BLD Pharm), poly(dialyldimethylammonium chloride) (PDDA), Mw ≈ 250,000 g/mol (Macklin, Shanghai, China), sodium polystyrene sulfonate (PSS), Mw ≈ 70,000 g/mol (Sigma-Aldrich, Steinheim, Germany).

The sotalol hydrochloride used in the study was purchased from Sigma-Aldrich (Germany). Sodium azide used in the immersion experiment was purchased from Dia-m (Moscow, Russia).

In capillary electrophoresis, the following reagents were used to prepare buffers and background electrolytes: sodium dihydrogen phosphate (Vekton, Russia), sodium hydroxide (Reakhim, Moscow, Russia), hydrochloric acid (Reakhim, Russia), phosphoric acid (Reakhim), 2-hydroxypropyl-β-cyclodextrine (HP-β-CD) (Sigma-Aldrich), and 18-crown-6 (Sigma-Aldrich, Germany). In liquid chromatography, the following solvents were used: water (LC-MS grade) and acetonitrile (LC-MS grade) obtained from Biosolve (Dieuze, France).

All reagents were used without additional purification. All aqueous solutions were prepared from deionized water with a resistivity of 18.2 MΩ·cm (Millipore, Molsheim, France). A stock solution of sotalol (1.0 mg/mL) was prepared by dissolving 10.0 mg pure sotalol hydrochloride in 10 mL of deionized water. A working solution at a concentration of 1 mM was obtained by diluting the stock solution 1:2.24 with deionized water. The stock solution was stored in a refrigerator at a temperature of −18 °C for 3 months. The working solution was stored in a refrigerator at a temperature of +4 °C for 1 week and was used for preparing calibration solutions and in immersion experiments.

### 3.2. Methods

#### 3.2.1. Capillary Electrophoresis

Electrophoretic experiments were performed using a capillary electrophoresis system, Capel-205 (Lumex, St. Petersburg, Russia), equipped with a spectrophotometric detector (190–400 nm) and a water-cooling cassette (±0.1 °C) for a bare fused-silica capillary (50 mm (id) × 375 mm (od)). The length of the used capillary to the detector was 53 cm. Processing was carried out using Elforan-205 software, version 4.2.5 (Lumex, Russia). A capillary was flushed with acetonitrile for 3 min at 2000 mbar, with distilled water for 2 min at 2000 mbar, and was conditioned with background electrolyte for 5 min at 2000 mbar before and between analyses. Background electrolytes consisted of 100 mM NaH_2_PO_4_ (pH 2) and 5 mM HP-β-CD or 25 mM HP-β-CD. 18-crown-6 was used in the concentration range of 0–10 mM in the background electrolyte for the separation of enantiomers of product **1**.

The CE methodology for the determination of sotalol was partially validated to ensure the reliability of the results. Background electrolyte consists of 100 mM phosphate solution (pH 2, adjusted by 1 M orthophosphoric acid) and 25 mM HP-β-CD. A working voltage was +20 kV. A working temperature was +25 °C. Spectrophotometric detection was performed at λ = 230 nm. A sample injection was hydrodynamic 5 s × 50 mbar.

Calibration solutions were prepared over a concentration range of 0.005 to 0.2 mM through serial dilution of the working solution (1 mM) to the desired concentrations. Each of them was injected into the capillary 3 times. Peak areas were recorded to form calibration curves. Calibration curves were constructed for total sotalol (sum of the areas of the R- and S-enantiomers) by plotting peak area against the respective sotalol concentration.

Calibration was carried out using 8 points. For each point, 3 measurements were made. The data were averaged and calibration curves calculated. Evaluated methods are linear between 0.005 and 0.2000 mM for sotalol. The calibration curves were represented by the following linear regression equations: y = 1.72 × 10^−3^x − 1.4 (R^2^ = 0.9994). The limit of quantitation (LOQ) was determined based on a signal-to-noise ratio. The LOQ was defined as the lowest concentration of sotalol that produced a peak with an S/N ratio of 10:1. This was verified by six replicate injections of the standard solution at the estimated LOQ level to ensure acceptable precision (RSD < 20%). LOQ was 0.005 mM for total sotalol and 0.0025 mM for each enantiomer. The limit of detection was defined as the lowest concentration of the analyte that produced an S/N ratio of 10:1. The LOD was experimentally verified by analyzing progressively diluted standard solutions of sotalol until the signal height reached three times the baseline noise level (*n* = 6).

The precision of the CE assay was evaluated by injecting a series of standard solutions at 3 concentration levels (0.025, 0.10, and 0.15 mM). The solutions containing sotalol were analyzed 5 times on the same day. Inter-day precision was assessed by analyzing the identical solutions on 3 consecutive days. Precision was expressed as the percentage relative standard deviation (% RSD) of sotalol total peak area. The intraday precision for sotalol, expressed as RSD, was 2.90 and 1.20% at the lowest and the highest concentrations. The respective values for the interday precision were 3.0 and 1.4%. The accuracy of the method was proved by the determination of the solution at 3 levels of total sotalol concentration (0.025, 0.1, and 0.15 mM). Determination was repeated 5 times. Accuracy was expressed as an average percentage, calculated as the ratio of the found concentration to the nominal concentration at each level. Selectivity was calculated in the resolution (R_s_) term based on the separation of the nearest product **2** from sotalol enantiomers. In blank solution (deionized water), no signal was observed for the analytical run. Enantioselectivity was calculated as the ratio of the migration times of the sotalol enantiomers. Additionally, the relative standard deviation of migration times for sotalol enantiomers was evaluated within days, between days, and from capillary to capillary. All results are presented in Table 1.

Each analytical batch commenced with the injection of a deionized water blank to eliminate potential carry-over effects. This was followed by analysis of a sotalol working standard diluted 10 times (0.1 mM) and, subsequently, the experimental samples. For all quantitative evaluations (with the exception of sorption kinetic studies), relative peak areas were employed. This normalization strategy was implemented to compensate for instrumental response fluctuations and to ensure that the analytical data remained robust and independent of absolute peak area variations across different capillaries and inter-day sessions.

#### 3.2.2. High-Performance Liquid Chromatography

A HPLC system (Shimadzu, Kyoto, Japan) with LC-30 AD binary pump, SPD-M20A diode-array detector, SIL-30AC cooling autosampler, and CTO-20A thermostat coupled to LCMS-8030 triple quadrupole mass-spectrometer was used for the identification of compounds. A chromatographic column, Inspire 3 µm Phenyl-hexyl, 150 × 2.1 mm from Dikma (Beijing, China), was utilized, operating at T = 45 °C. The eluents consisted of two mobile phases: mobile phase A consisted of water, and mobile phase B consisted of acetonitrile. The gradient profile was as follows: holding for 5 min on 5% phase B, followed by 5.0–15.0 min from 5 to 60% of mobile phase B, then by 15.0–17.0 min from 60% to 90% of mobile phase B, and holding for 6.0 min on 90% mobile phase B for washing the column. This was succeeded by a gradient back to 95% mobile phase A and 5% mobile phase B from 23.0 to 23.1 min to re-equilibrate. Total analysis run time was 25 min. The needle wash solvent consisted of 50% 2-propanol in water. The eluent flow rate was set at 0.300 mL/min. Liquid chromatography with mass spectrometric detection (HPLC-MS) was performed using an electrospray interface on a triple quadrupole mass spectrometer. The instrument was operated in positive-ion mode, with a spray voltage set at 4.50 kV. The temperature of nitrogen and flow rate were set to 400 °C and 10 L/min, respectively. Identification was conducted in scanning mode over the range 50–800 *m*/*z*. Nitrogen was used as a collision gas.

#### 3.2.3. Absorption Spectroscopy

Ultraviolet–visible (UV–Vis) absorption spectra were recorded on a UV-1800 spectrophotometer (Shimadzu, Japan). All spectra were measured at room temperature in a quartz cell with an optical pathway of 1 cm and a spectral resolution of 1 nm.

#### 3.2.4. Scanning Electron Microscopy

Scanning electron microscopy (SEM) images were obtained with a Zeiss Merlin microscope (Carl Zeiss, Oberkochen, Germany) at an accelerating voltage of 15 kV. SEM images were taken from at least three random domains. In order to prepare the samples for SEM measurements, 10 µL of GNP dispersion was drop-cast onto a polished silicon wafer and air-dried.

#### 3.2.5. Dynamic and Electrophoretic Light Scattering

Dynamic light scattering (DLS) was performed on a Zetasizer Nano ZS (Malvern Instruments Ltd., Malvern, UK). The refractive index value of 0.27 was used for gold, the viscosity of the solvent was set to 0.887 cP for water, and the refractive index was set to 1.33. Zeta potentials were measured in an electrophoretic light scattering regime using a cell with gold electrodes.

#### 3.2.6. Nanoparticle Tracking Analysis

Nanoparticle tracking analysis (NTA) was performed using the NP Counter instrument (NP Vision, Moscow, Russia). The instrument is equipped with an optical microscope objective lens of 20× magnification, a laser diode with a wavelength of 637 nm and a maximum power output of 30 mW, and a RisingCam YW500 complementary metal-oxide-semiconductor camera. The camera was operated at 15 frames per second.

### 3.3. Gold Nanoparticle Synthesis and Modification

**Figure 10:**
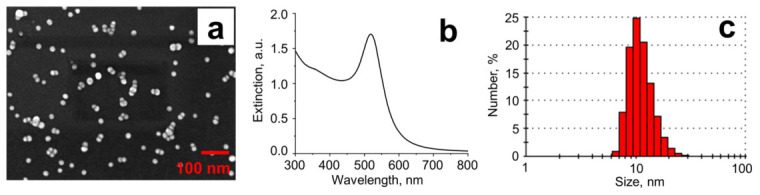
SEM image (**a**), extinction spectrum (**b**), and hydrodynamic size distribution histogram (**c**) of as-synthesized gold nanoparticles.

Spherical gold nanoparticles were synthesized by reducing Au^3+^ with sodium citrate using a known method [43]. A procedure consists of heating 50 mL of a 1 mM HAuCl_4_ solution to boiling under reflux with continuous stirring. Next, 5 mL of 38.8 mM sodium citrate solution is added to the boiling mixture. After the mixture changed color to dark red, boiling continued for 15 min. The extinction spectrum of the obtained GNP dispersion is characterized by a band centered at 520 nm. SEM showed a nanoparticle size of 12 ± 2 nm, which is in good agreement with an average size of 11.4 ± 3.2 nm revealed by dynamic light scattering (Figure 10). The molar concentration of nanoparticles in the obtained dispersion, measured by nanoparticle tracking analysis, is (1.8 ± 0.2) × 10^−8^ M.

**Figure 11:**
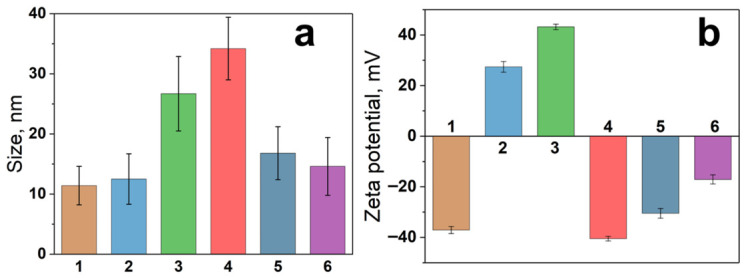
Hydrodynamic size (**a**) and zeta potential (**b**) of bare and modified gold nanoparticles: cGNP (1), GNP@CTAB (2), GNP@PDDA (3), GNP@PDDA@PSS (4), GNP@PEG-SH (5), GNP@L-Cys (6).

Functionalization of bare citrate-stabilized GNPs (cGNPs) with L-cysteine was performed by adding a 10 mM stock solution of the amino acid to the cGNP dispersion in a volume ratio of 1:9. The surface modification of cGNPs by surfactants and polyelectrolytes was carried out according to a general procedure developed in our previous study [44]. Briefly, the nanoparticle dispersion was added dropwise to a solution of modifier in a volume ratio of 1:1. A concentration of CTAB solution was 0.02 M, and PEG-SH, PDDA, and PSS were used as solutions with a concentration of 2 g/L. After stirring for 2 h, the mixture of GNP and modifier was centrifuged for 40 min at 15,000 rpm, and the separated particles were redispersed in a volume of deionized water equal to the original volume. The success of nanoparticle functionalization was verified by the measurement of hydrodynamic size and zeta potential (Figure 11). An increase in hydrodynamic size was observed for each modifier; it was most significant for the polymers and minor for small modifiers such as CTAB or L-cysteine. However, the recharging of the surface in the case of CTAB and the significant decrease in the zeta potential value in the case of L-cysteine allow us to conclude that effective surface modification has occurred.

### 3.4. Immersion Experiments

Immersion experiments were performed in the following way. A solution of sotalol with a concentration of 1 mM was added to a dispersion of bare or modified GNP in a volume ratio of 1:9. The obtained mixture was incubated at room temperature for various periods of time, then centrifuged at 18,000 rpm for 30 min. Further, a supernatant was collected and used for analyses without any additional treatment.

Based on the known molar concentration of nanoparticles in the dispersion ((1.8 ± 0.2) × 10^−8^ M) and their average size (12 nm), a number of sotalol molecules per m^2^ of gold surface was estimated as 1.2 × 10^19^ molecules per m^2^, which is equivalent to 20 μM per m^2^.

To adjust the required pH of the cGNP dispersion, small amounts of 1 M nitric acid or 0.1 M sodium hydroxide were used, with a control by triple-zone indicator paper. In the immersion experiment with sodium azide, its solution (0.01–5 M) was added to the GNP and sotalol mixture at a volume ratio of 1:9. Immersion experiments were performed under standard conditions, without special protection from light. The stability of sotalol itself in these conditions during the period required for each experiment was verified every time by analyzing a control sample.

The immersion experiment under oxygenation was performed by passing oxygen gas through a dispersion of cGNPs in sotalol for 10 min, followed by keeping the mixture in a sealed vessel for 24 h. The immersion experiment under deoxygenation was performed in a second way. The dispersion of cGNPs with sotalol was saturated with argon, sonicated, and vacuumed in a sealed vessel repeatedly three times; after that, it was left for 24 h.

## 4. Conclusions

In summary, multicenter oxidation of sotalol was revealed on the surface of citrate-stabilized gold nanoparticles. The reliably identified by CE and HPLC-MS products showed that sotalol undergoes N-dealkylation coupled with hydroxyl group oxidation, yielding a ketone with a primary amino group. The experiments performed at deoxygenation and with radical scavenger additive showed that the chemisorbed oxygen, which is activated on the surface into reactive oxygen species, plays a key role in sotalol oxidation. Control experiments using hydrogen tetrachloroaurate and sodium citrate solutions confirmed the catalytic effect of gold nanoparticles. Apparently, the surface oxidation of both functional groups of sotalol proceeds through a single-electron transfer mechanism. GNP-assisted oxidation of the hydroxyl group in the propanolamine moiety of sotalol, distinguishing it from the previously studied beta blocker propranolol, occurs due to stabilization of its transition state by the benzyl alcohol fragment.

The study demonstrates the importance of engagement of direct methods for the separation and identification of structurally similar compounds in investigations of GNP-assisted reactions. The applied approach enabled us to discern three products of the surface reaction, which would be extremely difficult to achieve if the solution phase were neglected. It should be noted that chemical transformations occur in a widely used drug; therefore, the study emphasizes the need for careful use of plasmonic materials in combination with bioactive molecules, in particular as drug carriers or non-destructive sensors.

The revealed transformation of sotalol on the GNP surface may have significant clinical implications. The loss of the N-isopropyl group, essential for beta-adrenergic receptor recognition, likely results in reduced therapeutic efficacy. While toxicity data for the revealed sotalol oxidation products are limited, the formation of reactive carbonyls or primary amines can potentially increase the risk of non-specific interactions with cellular components. Thus, monitoring drug transformation in the presence of metallic nanoparticles is important to ensure patient safety.

While this study provides mechanistic insights, several limitations must also be considered. The in vitro conditions used do not fully reproduce the complex physiological environment. Factors such as the presence of plasma proteins, blood flow, and homeostatic pH buffering can modulate the catalytic activity of gold nanoparticles. Furthermore, the gold nanoparticle concentrations used in this study were significantly higher than those typically used in in vivo studies.

From a drug delivery perspective, our results highlight the necessity of applying biocompatible coatings to physically shield the drug substances from the catalytic gold core and engineering delivery vehicles that can maintain local pH levels to prevent pH-dependent formation of degradation products.

Overall, this study underscores the need for a rigorous analytical approach to evaluate drug-nanoparticle interactions, as understanding these catalytic transformations is paramount to ensuring the safety and therapeutic integrity of nanomedicine.

## Figures and Tables

**Table 1:** Validation parameters of capillary electrophoresis.

Parameter	Value			
Linearity	0.005–0.2 mM			
LOQ	0.005 mM			
LOD	0.0016 mM			
		0.025 mM	0.100 mM	0.150 mM
Accuracy (RSD, %)	Intra-day (*n* = 5)	99.8	98.2	98.4
	Inter-day (*n* = 15)	99.9	98.7	98.0
Precision (RSD, %)	Intra-day (*n* = 5)	2.9	2.0	1.2
	Inter-day (*n* = 15)	3.0	1.9	1.4
Precision of migration time (RSD, %)	Intra-day (*n* = 5)		2.7	
	Inter-day (*n* = 15)		3.6	
	Capillary-to-capillary reproducibility (*n* = 5)			
	8.3			

## Data Availability

The original contributions presented in this study are included in the article. Further inquiries can be directed to the corresponding author.

## Abbreviations

The following abbreviations are used in this manuscript:

GNP	Gold nanoparticle
cGNP	Citrate-stabilized gold nanoparticle
CTAB	Cetyltrimethylammonium bromide
PDDA	Poly(dialyldimethylammonium chloride)
PSS	Sodium polystyrene sulfonate
L-Cys	L-cysteine
PEG-SH	Mercapto polyethylene glycol
HP-β-CD	2-Hydroxypropyl-β-cyclodextrine
CE	Capillary electrophoresis
HPLC	High-performance liquid chromatography
UV–Vis	Ultraviolet–visible
SEM	Scanning electron microscopy
DLS	Dynamic light scattering
NTA	Nanoparticle-tracking analysis
ROS	Reactive oxygen species
